# Effects of Calcium Chloride Crosslinking Solution Concentration on the Long-Term Cell Viability of 16HBE14o- Human Bronchial Cells Embedded in Alginate-Based Hydrogels

**DOI:** 10.3390/biomimetics10010040

**Published:** 2025-01-10

**Authors:** Nathan Wood, Esther I. Doria, Taieba Tuba Rahman, Wanhe Li, Zhijian Pei, Hongmin Qin

**Affiliations:** 1 Department of Biology, Texas A&M University, 3258 TAMU, College Station, TX 77843, USA; edoria@tamu.edu (E.I.D.); wli01@bio.tamu.edu (W.L.); 2 Department of Industrial & Systems Engineering, Texas A&M University, 3127 TAMU, College Station, TX 77843, USA; taieba_tuba@tamu.edu (T.T.R.); zjpei@tamu.edu (Z.P.)

**Keywords:** 3D culture, alginate, crosslinking, epithelia, methylcellulose, viability

## Abstract

In this preliminary study, the long-term effects of calcium chloride crosslinking concentration on viability of 16HBE14o- human bronchial epithelial cells embedded in alginate-extracellular matrix (ECM) or alginate–methylcellulose–ECM hydrogels have been investigated. There is currently a limited understanding regarding the effects of crosslinking solution concentration on lung epithelial cells embedded in hydrogel. Furthermore, the effects of calcium chloride concentration in crosslinking solutions on other cell types have not been reported regarding whether the addition of viscosity and stiffness tuning agents such as methylcellulose will alter the responses of cells to changes in calcium chloride concentration in crosslinking solutions. While there were no significant effects of calcium chloride concentration on cell viability in alginate–ECM hydrogels, there is a decrease in cell viability in alginate–methylcellulose–ECM hydrogels crosslinked with 300 mM calcium chloride crosslinking solution. These findings have implications in the maintenance of 16HBE14o- 3D cultures with respect to the gelation of alginate with high concentrations of ionic crosslinking solution.

## 1. Introduction

The process of 3D bioprinting entails the depositing of biomaterials known as bioinks to fabricate a particular structure for application in tissue engineering, drug delivery, or 3D cell culture [1]. Regarding 3D cell culture, bioinks embed animal, plant, fungal, or bacterial cells in a spatial orientation that is more similar to their native environment, allowing for researchers to gain an understanding of drug responses, signal transduction, and differentiation [2]. However, bioprinting, including extrusion bioprinting which is dependent on pneumatic pressure applied upon the bioink, requires a bioink that both maintains healthy cells, and retains physical properties that make printing feasible [3].

Derived from brown algae, sodium alginate and its derivatives are a popular bioprinting component due to availability, biocompatibility both in vivo and in vitro, tunable viscosity, and ability to covalently or ionically crosslink to form hydrogels [4]. The polymer structure of alginate is responsible for its ability to form hydrogels in the presence of divalent cations. Alginate consists of consecutive monomeric units of (1 → 4)-β-D-mannuronate sugars known as M-blocks, consecutive monomeric units of ɑ-L-guluronate sugars known as G-blocks, and alternating sequences of both (1 → 4)-β-D-mannuronate, and ɑ-L-guluronate known as MG-blocks [4,5]. Upon exposure to cations such as calcium, the carboxyl groups of the alginate G-blocks interact with the cation with a high affinity. This interaction alters the otherwise linear conformation of alginate polymers into an “egg-box” conformation, rapidly resulting in a sol-gel reaction and turning into a water-retentive hydrogel [6,7,8,9]. This compactness is observed by reduced swelling or retention of water, which is the result of conformation changes brought about by the interactions of the cation ions and G-block carboxyl groups [4,6,8].

Calcium chloride (CaCl_2_) solutions are often employed as an ionic crosslinking agent, with Table 1 summarizing reported studies that employed calcium chloride for ionic crosslinking of alginate-based hydrogels [6,10,11,12,13,14,15]. However, the presence of high levels of calcium ions can harm cells. Cao et al. observed that Schwann cells experience a decline in both viability and proliferation as calcium chloride concentration and crosslinking time increase [16]. Crosslinking solutions can potentially alter cell behavior by transiently increasing osmolality to an inappropriate level [17]. A 300 mM calcium chloride solution has an osmolality of approximately 900 mOsm kg^−1^, as calcium chloride in water dissociates into one divalent calcium osmolyte, and two monovalent chloride osmolytes. Comparatively, human plasma has an osmolality that approaches 290 mOsm kg^−1^, and the Sigma Aldrich Modified Eagle Medium (MEM, M2279) used for cell culture has a manufacturer-defined osmolality range between 281 and 311 mOsm kg^−1^ [17,18,19]. Furthermore, many cellular processes operate in a calcium dependent manner, which may or may not become disordered if excessive calcium is suddenly introduced then removed in the culturing environment [20,21]. As certain cell types are more resilient to high osmolality levels than others, responses of cells to changes in the concentration of ionic crosslinking solutions need to be determined empirically.

In bioinks, alginate is often not the only component. Soluble cellulose derivatives such as methylcellulose are frequently added to alginate solutions to increase the viscosity of a bioink for 3D bioprinting [22]. Previously Li et al. evaluated the bulk rheological characteristics of a 3% (*w*/*v*) alginate, and alginate–methylcellulose mixtures at ratios of 1:3, 3:3, and 3:9 (% *w*/*v*) for extrusion bioprinting. They observed greater than 95% cell viability when L929 mouse fibroblast cells were incorporated between layers of the 3:9% alginate–methylcellulose bioink [14]. Additionally, Duin et al. successfully printed a 3:9 (% *w*/*v*) alginate–methylcellulose hydrogel laden with mouse pancreatic islets, achieving 60–80% cell viability over seven days [15]. However, alginate–methylcellulose hydrogels have yet to be explored for use in the culturing of lung epithelial cells to develop spheroids for further biological experimentation.

The 16HBE14o- cell is an immortalized line of human bronchial epithelial cells. It originated from the healthy lung tissue of a 1 year old male and was immortalized in vitro using an expression vector for the SV40 Large T Antigen, altering pathways associated with cell death and proliferation [23,24,25]. The 16HBE14o- cell distinguishes itself from lines originating from patient derived cancer cells such as A549 in that 16HBE14o- not only lacks the genetic mutations associated with carcinomas extracted from patients, but also lacks the epigenetic history that results from a patient’s lifelong activities, such as tobacco use [26,27]. Because of these characteristics, the 16HBE14o- human bronchial epithelial cell line is an excellent immortalized model for the study of the cell biology of the lung epithelial layer through the use of 3D bioprinting techniques. However, little work has been completed regarding the use of 16HBE14o- cells in 3D bioprinting of alginate hydrogels, and more work needs to be done to successfully employ bioprinting as a method to improve the scalability and complexity of 3D culturing.

This study explored if there is a delayed effect of calcium chloride crosslinking solution on the viability of 16HBE14o- human bronchial epithelial cells embedded in hydrogels consisting of either sodium alginate or both sodium alginate and methylcellulose. Both alginate and alginate–methylcellulose solutions include extracellular matrix (ECM) components by using a tissue culture flask coating solution as a solvent. The cell-laden solutions were then ionically crosslinked by bathing in 100 mM or 300 mM calcium chloride solutions for five minutes to form hydrogels.

## 2. Materials and Methods

### 2.1. Experimental Design

The 16HBE14o- cells with a density of approximately 2.03 × 10^5^ cell mL^−1^ were embedded in two types of solutions: alginate–ECM solution, or alginate–methylcellulose–ECM solution. Each of these cell-containing solutions were deposited into a 6-well plate with its own hydrogel composition and crosslinking solution concentration, followed by experiment time point for a total of 36 samples across 6 6-well plates. Both solutions were crosslinked using crosslinking solutions with two different calcium chloride concentrations: a 100 mM calcium chloride solution, or a 300 mM calcium chloride solution. This resulted in four independent groups: alginate–ECM hydrogels crosslinked with 100 mM calcium chloride, alginate–ECM hydrogels crosslinked with 300 mM calcium chloride, alginate–methylcellulose–ECM hydrogels crosslinked with 100 mM calcium chloride, and alginate–methylcellulose–ECM hydrogels crosslinked with 300 mM calcium chloride. All four experimental groups were performed in triplicate.

### 2.2. Cell Culture

The 16HBE14o- cells were purchased from Sigma Aldrich (Product No. SCC150, St. Louis, MO, USA) in January 2024. Cells used in this experiment were no older than their seventh passage since receipt from Sigma Aldrich. The 16HBE14o- cell was cultured in complete Modified Eagle Medium (MEM, Sigma Aldrich M2279) with supplemental 2 mM glutamine and 10% (*v*/*v*) Fetal Bovine Serum (Corning). To mitigate cryptic contamination, cells were cultured without antimicrobials from thawing until washout of crosslinking solution from hydrogels. After washout of crosslinking solution, cell-laden hydrogels were cultured in complete MEM with penicillin (100 U mL^−1^), streptomycin (100 µg mL^−1^), and amphotericin B (0.25 µg mL^−1^). Cells subsampled from those that were embedded in hydrogels were cultured as traditional monolayers on a glass coverslip, and tested negative for mycoplasma contamination, demonstrated via a lack of extranuclear fluorescence using Hoecsht 33342 (Product No. H3570, Thermo Fisher Waltham, MA, USA) [18].

### 2.3. Reagents for Hydrogels

Both sodium alginate (15–25 cP, 9005-38-3) and methylcellulose (4000 cP, 9004-67-5) were purchased from Sigma Aldrich.

Extracellular matrix (ECM) consists of Bovine Serum Albumin (BSA, 100 µg mL^−1^, Sigma Aldrich 126575), Advanced Biomatrix PureCol Bovine Type I Collagen (30 µg mL^−1^, Sigma Aldrich 5006), and Human Fibronectin (10 µg mL^−1^, Sigma Aldrich F2006-5MG). The ECM components were prepared in accordance with Sigma Aldrich protocols provided in the vendor’s 16HBE14o- data sheet [23]. For alginate and alginate–methylcellulose solutions, two 25 mL volumes were placed in sterile glass bottles with a magnetic stir bar with penicillin (100 U mL^−1^), streptomycin (100 µg mL^−1^), and amphotericin B (0.25 µg mL^−1^) being added to the ECM solution for future use as the alginate–ECM and alginate–methylcellulose–ECM solutions.

**Figure 1:**
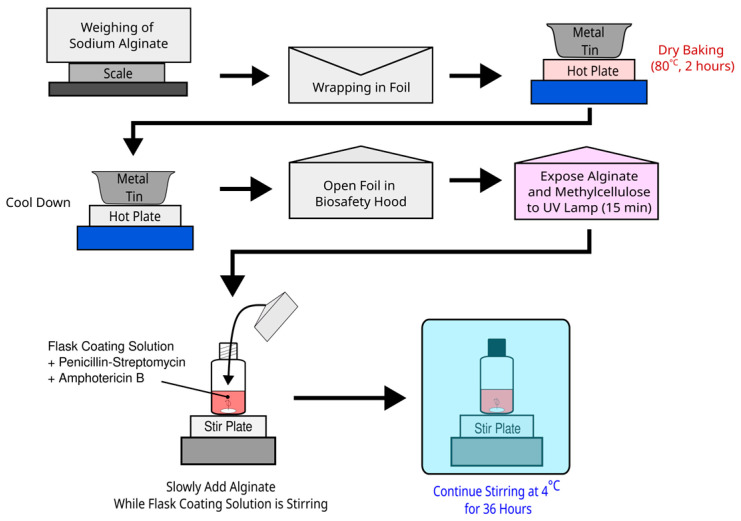
Preparation of alginate–ECM hydrogel. Alginate–methylcellulose–ECM hydrogel solutions are prepared in the same manner, but with methylcellulose added after the addition of sodium alginate.

Figure 1 details how both solutions were prepared. To maintain sterility, alginate powder was weighed, wrapped in aluminum foil, and then placed in a metal tin, with additional folded aluminum foil at the bottom of the tin to keep the alginate from directly contacting the bottom. This tin was then placed on a hot plate set to approximately 80 Celsius for 2 h. After being allowed to cool to room temperature, the tin was brought to a biosafety hood, and the alginate powder was carefully unwrapped on the working surface. The tin was removed, and the powder was exposed to the UV lamp of the biosafety hood for no more than 15 min [28]. After this, the prealiquoted flask coating solutions were placed in the biosafety hood. The stir plate was turned on and set to a mild cycle based on the qualitative gauge of the stir plate, and alginate was slowly added to the solution for about 5 min. Once addition was complete, the alginate–ECM solution was secured and was stirred at a similar cycle in a 4 °C cold room for 36 h. For all experiments, a 4% (*w*/*v*) alginate–ECM solution was prepared by adding 1 g of sodium alginate to 25 mL of ECM-containing MEM solution. To produce alginate–methylcellulose–ECM solution, methylcellulose was added to the flask coating solution shortly after the addition of sodium alginate. Methylcellulose was added more carefully for about 10 min as it greatly increased the viscosity of the solution, requiring a more aggressive stirring over 36 h. The final concentration of methylcellulose was 4% (*w*/*v*), a 1:1 ratio with dissolved sodium alginate.

### 2.4. Preparation of Cell-Laden Hydrogels

Cells on the surface of culture flasks were detached via trypsin and then centrifuged down at approximately 1300× *g* for 4 min. Cells were resuspended in MEM without antimicrobials. A 100 µL aliquot of resuspended cells was diluted in 900 µL phosphate buffered saline (1X PBS) with an additional 10 µL of trypan blue dye (0.4%) to determine cell viability. The resuspended cell density and viability were assessed according to the manufacturer’s protocol using the Nexcelom T4 Auto Cellometer (Revvity, Waltham, MA, USA). Resuspended cells were found to be 100 percent viable. A total of 1 mL of resuspended cells were added to 2 mL of alginate–ECM or alginate–methylcellulose–ECM solutions for a final cell density of approximately 2.03 × 10^5^ cell mL^−1^. Cells were then mixed into the solution using an autoclaved metal spatula. A total of 100 µL of cell-laden alginate–ECM solution was then pipetted into well-plate wells cross-linked for 5 min in either 100 mM or 300 mM aqueous calcium chloride. About 100–250 µL of alginate–methylcellulose–ECM was added into individual well plate wells using an autoclaved metal spatula with a 3.5 mm tapered end, and then crosslinked. Crosslinking was performed in a delayed manner to avoid overexposure to the crosslinking solution while managing multiple samples. Samples awaiting crosslinking were carefully stored in a 37 °C, 5% CO_2_ incubator.

### 2.5. Measurement of Cell Viability

**Figure 2:**
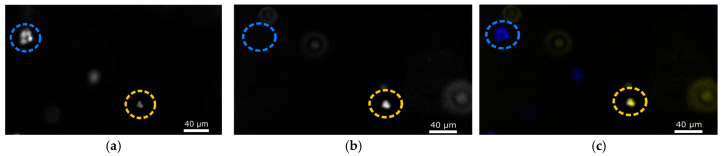
Use of NucBlue Live and NucGreen Dead to determine cell viability in hydrogels. In all subfigures, living cells are circled in blue, whereas dead cells are circled in yellow. (**a**) Staining of all nuclei using NucBlue Live. (**b**) Staining of the nuclei of dead and dying cells using NucGreen Dead. (**c**) A merged image of both the DAPI (NucBlue) and FITC (NucGreen) channels.

A nucleus fluorescence-based viability assay was conducted using an Invitrogen ReadyProbes^TM^ cell viability imaging kit (R37609, Thermo Fisher Waltham, MA, USA). NucBlue Live should stain all cell nuclei and result in fluorescence under UV excitation typically provided by a DAPI compatible filter, whereas NucGreen Dead should stain nuclei but can only permeate dead and dying cells and should excite under a FITC compatible filter (Figure 2). Cells were imaged using an Echo Revolution microscope in its inverted configuration, with a 37 °C incubator mounted to the stage and supplied a 5% CO_2_ gas mixture via a Live Cell Instruments FC-9 gas mixer. At least six images of a single focal plane at different Z positions were taken using Chroma FITC and DAPI fluorescence filters. Cell viability is determined by the ratio of green nuclei to blue nuclei, shown in Equation (1). Percent viability per individual hydrogel sample is then derived from the averaged viability of all images taken of that sample.

(1) $$Viability\left(\%\right)=\left(1-\frac{Green\;Nuclei}{Blue\;Nuclei}\right)\times 100$$

The normality of the observed cell viabilities was tested using the Shapiro–Wilks Test (lowest *p* = 0.0995). Student’s Two Tail *t*-Test was used to compare the mean percent viability determined on Days 3, 6, and 9 of hydrogels cross-linked using either 100 mM or 300 mM calcium chloride solution. *p* values below 0.05 were considered significant. Data are represented as arithmetic means and standard deviations. Significance is reported as * (*p* < 0.05), and ** (*p* < 0.01).

### 2.6. Observation of Cell Clusters: Counting the Number of Cells Within Clusters

Hydrogels were washed with 1X PBS three times for one minute each, and fixed with fresh Paraformaldehyde (4% *w*/*v* in 1X PBS) for two hours, then washed again with 1X PBS three times for one minute each. Cells were then stained with SYTOX Green (Invitrogen) nuclei stain in a 1:30,000 dilution in 1X PBS for 30 min in a 37 °C 5% CO_2_ incubator. Samples were washed again with 1X PBS three times for one minute each, then carefully transferred to a cleaned microscope slide using an autoclaved metal spatula.

**Figure 3:**
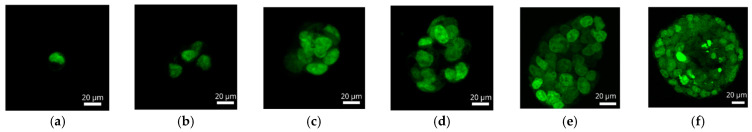
Cell clusters are categorized from the number of cells within them, determined using 3D reconstructions of confocal Z stacks. (**a**) Single cells were incidentally found and were recorded, despite not being clusters of cells. (**b**) Clusters of 2–4 cells. (**c**) Clusters of 5–8 cells. (**d**) Clusters of 9–12 cells. (**e**) Clusters of 13–24. (**f**) Clusters of more than 24 cells.

Samples were then imaged using a Zeiss Imager.2 microscope with LSM800 laser scanning confocal unit with a 20X/0.75 dry objective. Cell aggregates were first identified via phase contrast and then the LSM800’s 488 nm excitation laser was used to take a Nyquist-rated (0.53 µm) Z stack. The upper and lower optical sections of the confocal Z stack were determined when the aggregates become completely out of focus. Incidentally, single cells were observed alongside clusters of cells and were also recorded. Cells in each cluster were counted manually from a 3D reconstruction of the Z stack accomplished using a plugin intrinsic to FIJI is Just ImageJ (FIJI) version 1.54k. Cell clusters, including the single cells observed were then categorized as having either one cell, 2–4 cells, 5–8 cells, 9–12 cells, 13–24 cells, or more than 24 cells per aggregate, as shown in Figure 3. Fisher’s Exact Test was used to determine if there was an association between cross-linker concentration and the prevalence of clusters within the cell count categories. *p* values below 0.05 were considered significant. Data are represented as percentages of the total observed clusters and single cells.

### 2.7. Observation of Cell Clusters: Assessing the Spatial Confinement of Cells Within Clusters

Clusters categorized per Section 2.6 had their volume approximated assuming the shape of a prolate spheroid (Equation (2)) [24]. The equatorial diameter a is determined by assuming that the maximum intensity projection of the aggregate is a circle, and finding the radius from the area measured in FIJI (Equation (3)). The polar diameter c is determined by multiplying the number of optical sections taken for the Z stack by the Nyquist Rate. To determine the spatial confinement, or the amount of cluster volume that each cell approximately has, the total volume, V, of the cluster determined using Equations (2) and (3) is divided by the number of cells in the cluster.

(2) $$V=\frac{1}{6}\pi a^{2}c$$

(3) $$a=2r=\sqrt{\frac{Area}{\pi }}$$

For statistical analysis, cell clusters with more than 8 cells were subsampled for statistical analysis, with single cells and clusters with either 2–4 or 5–8 cells being excluded. Spatial confinement of cell clusters, in units µm^3^ cell^−1^ were found to not be normally distributed per test group using the Shapiro–Wilks test (lowest *p* = 0.0026). Because of the non-normal distribution of some of the test groups, Wilcoxon’s Rank Sum Test was used to compare the spatial confinement of cell clusters found within hydrogels crosslinked with either 100 mM or 300 mM calcium chloride. *p* values below 0.05 were considered significant. Data are represented as arithmetic means and standard deviations. Significance is reported as * (*p* < 0.05), and ** (*p* < 0.01).

## 3. Results

### 3.1. Cell Viability in Alginate–ECM Hydrogels

**Figure 4:**
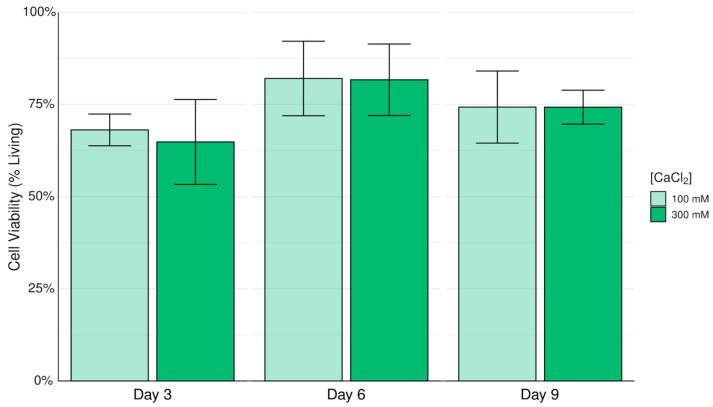
Viability of 16HBE14o- cells embedded in alginate–ECM hydrogels crosslinked with 100 mM or 300 mM calcium chloride solution. Data reported as arithmetic mean and standard deviation.

Figure 4 shows that there is no significant difference in 16HBE14o- cell viability between alginate–ECM hydrogels crosslinked with either 100 mM or 300 mM calcium chloride solution for 5 min. Therefore, the viability of 16HBE14o- cells, when embedded in an alginate–ECM hydrogel, is insensitive to the change in concentration of calcium chloride crosslinking solution ranged from 100 to 300 mM.

### 3.2. Cell Viability in Alginate–Methylcellulose–ECM Hydrogels

**Figure 5:**
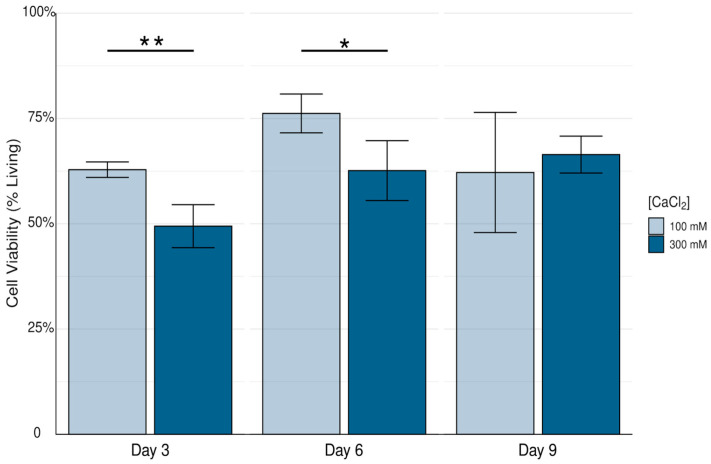
Viability of 16HBE14o- cells embedded in alginate–methylcellulose–ECM hydrogels crosslinked with 100 mM or 300 mM calcium chloride solution. Data reported as arithmetic mean and standard deviation. Significance is reported as * (*p* < 0.05), and ** (*p* < 0.01).

As shown in Figure 5, on both Day 3 (*p* = 0.0015) and Day 6 (*p* = 0.014), there was a significant decrease in 16HBE14o- cell viability when embedded in alginate–methylcellulose–ECM hydrogels and crosslinked with 300 mM calcium chloride solution. On Day 9 there was no significant difference in cell viability between hydrogels crosslinked with the two crosslinking solutions, potentially due to dividing cells allowing the hydrogel to recuperate from large amounts of cell death. This difference is striking compared to observations in alginate–ECM hydrogels (Figure 4).

### 3.3. Observation of Cell Clusters in Alginate–ECM Hydrogels

**Figure 6:**
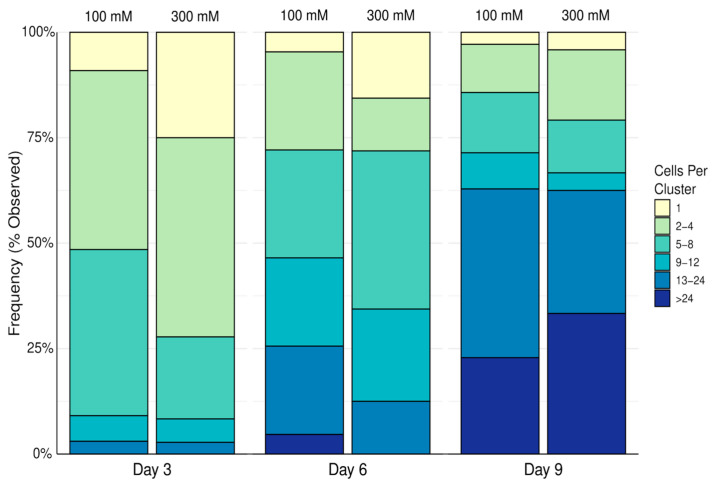
Frequency distribution of the 16HBE14o- cells in observed clusters in alginate–ECM hydrogels crosslinked with 100 mM or 300 mM calcium chloride solution. Data reported as a percentage of the total observed clusters and single cells.

Figure 6 demonstrates that over time, cell clusters bearing larger numbers of cells become more prevalent. It was found that there was no association between calcium chloride concentration and any differences in the frequency of a particular cluster’s cell count. These observations suggest that in an alginate–ECM hydrogel, calcium chloride concentration does not have any long-term effect on the ability for living cells to either continue dividing or aggregate together over time.

### 3.4. Observation of Cell Clusters in Alginate–Methylcellulose–ECM Hydrogels

**Figure 7:**
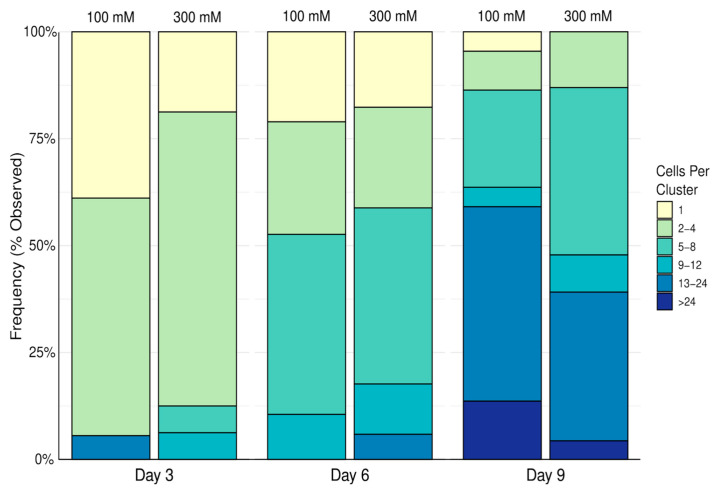
Frequency distribution of the 16HBE14o- cells in observed clusters in alginate–methylcellulose–ECM hydrogels crosslinked with 100 mM or 300 mM calcium chloride solution. Data reported as a percentage of the total observed clusters and single cells.

Figure 7 demonstrates that cell clusters with increasing numbers of cells become more prevalent over time. Similarly to the alginate–ECM hydrogels, it was found that there was no association between calcium chloride concentration and the frequency distribution of cell clusters based on cell count.

### 3.5. Spatial Confinement of Alginate–ECM Hydrogels

**Figure 8:**
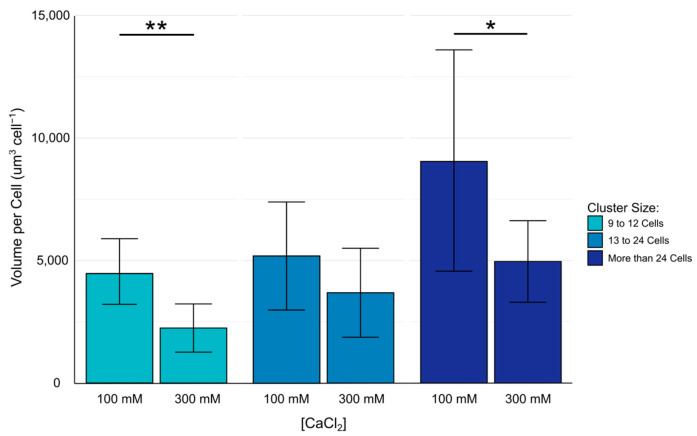
Volume per cell within 16HBE14o- cell clusters embedded in alginate–ECM hydrogels crosslinked with 100 mM or 300 mM calcium chloride solution. Data reported as arithmetic mean and standard deviation. Significance is reported as * (*p* < 0.05), and ** (*p* < 0.01).

Cell clusters of 9–12 (*p* = 0.002) and more than 24 cells (*p* = 0.02) within hydrogels crosslinked with 100 mM calcium chloride solution exhibited a higher volume per cell than hydrogels crosslinked in 300 mM calcium chloride solution (Figure 8). This suggests that higher concentrations of calcium chloride produce a hydrogel that confines cells into a tighter space. Conversely, cell clusters with 13–24 cells exhibited no significant difference in spatial confinement, an observation that will require additional experimentation to fully elucidate.

### 3.6. Spatial Confinement of Alginate–Methylcellulose–ECM Hydrogels

It should be noted that unlike alginate–ECM hydrogels, alginate–methylcellulose–ECM hydrogels crosslinked with 300 mM calcium chloride solution did not have enough clusters of more than 24 cells to perform hypothesis testing. Because of a lack of observations, clusters of more than 24 cells were excluded from analysis. This would also suggest that the cumulative stiffness of the presence of methylcellulose and calcium chloride solution may limit the population cell clusters can reach by a given time point.

**Figure 9:**
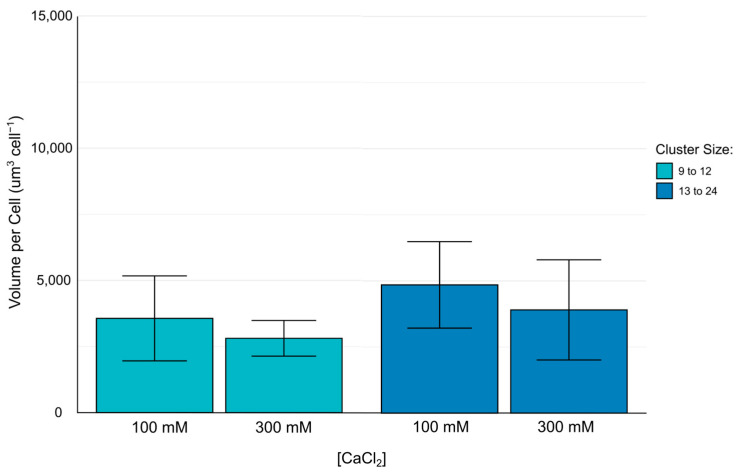
Volume per cell within 16HBE14o- cell clusters embedded in alginate–methylcellulose–ECM hydrogels crosslinked with 100 mM or 300 mM calcium chloride solution. Data reported as arithmetic mean and standard deviation.

Figure 9 demonstrates that there were no significant differences in the volume per cell in clusters of 9–12 and 13–24 cells in alginate–methylcellulose–ECM hydrogels. This result, in contrast to the differences observed in alginate–ECM hydrogels suggests that the presence of methylcellulose may cause an effect on spatial confinement of cells growing in clusters that is not further altered by calcium chloride concentration.

## 4. Discussion

In this study, the effects of the concentration of calcium chloride crosslinking solution on the long-term viability of 16HBE14o- cells embedded in either alginate–ECM or alginate–methylcellulose–ECM hydrogels were examined. From the observations at 3, 6, and 9 days after crosslinking, it was concluded that the viability of 16HBE14o- cells embedded in alginate–ECM hydrogels was unaffected to the change in concentration of calcium chloride crosslinking solution between 100 and 300 mM. The insensitivity to calcium chloride concentration between 100 and 300 mM indicated that the long-term viability of embedded 16HBE14o- cells was not affected by any increase in stiffness or decrease in porosity associated with alginate crosslinked with crosslinking solution of a higher calcium chloride concentration. Conversely, it was observed 3 and 6 days after crosslinking that 300 mM calcium chloride crosslinking solution did lead to a reduction in cell viability in alginate–methylcellulose–ECM hydrogels. The lack in difference in viability of 16HBE14o- cells at different levels of calcium chloride concentration in alginate–ECM and observed difference in viability in alginate–methylcellulose–ECM hydrogels suggest that the presence of methylcellulose in hydrogels likely affects the viability of 16HBE14o- cells.

The compactness of cells in clusters has also been investigated for both alginate–ECM and alginate–methylcellulose–ECM hydrogels crosslinked with either 100 mM or 300 mM calcium chloride solution. While determining the exact volume of a cell cluster is challenging due to the use of only a nuclei stain instead of membrane or cytoplasmic stains, the volume per cell, based on the number of nuclei in a cluster, provides a reasonable approximation of spatial confinement. This approximation is based on the rationale that nuclei confined to a smaller space will likely correspond with more spatially confined cells [29]. In alginate–ECM hydrogels crosslinked with 300 mM calcium chloride solution, clusters of nine to twelve cells and clusters of more than 24 cells were more spatially confined than the cells embedded in alginate–ECM hydrogels crosslinked with 100 mM calcium chloride solution. This observation is consistent with the reported result that increased calcium levels resulted in a more compact and less porous hydrogel [29]. However, the results from this study did not show significant difference in spatial confinement of clusters of 13 to 24 cells in alginate–ECM hydrogels, an observation that requires additional investigation. There were no significant differences in cluster spatial confinement between alginate–methylcellulose–ECM hydrogels crosslinked with 100 mM and 300 mM calcium chloride solutions. The observed lack of significant differences in volume per cell in alginate–methylcellulose–ECM hydrogels was likely attributable to methylcellulose contributing significantly to the hydrogel’s stiffness, effectively overshadowing the contribution from calcium–alginate crosslinking. This interpretation is consistent with the established role of methylcellulose in enhancing the viscosity of alginate solutions prior to crosslinking [14,22].

Further investigation into the compactness of cells in clusters will require assessment of alteration in physical properties to both alginate–ECM and alginate–methylcellulose–ECM hydrogels post-crosslinking via compression yield testing [30]. This is particularly important given that stiffer hydrogels can influence cell growth over time, in addition to altering cell behavior [31,32]. Furthermore, this study did not characterize how calcium chloride concentration affects solute diffusion in crosslinked hydrogels, which may impact cell performance in stiffer hydrogels. Future experiments will need to characterize the porosity of crosslinked hydrogels. Such characterization has been reported in the literature, where electron microscopy was used to determine hydrogel porosity and fluorescence recovery after photobleaching (FRAP) microscopy was used to approximate solute diffusion [8,33,34,35,36].

## 5. Conclusions

This study has revealed that the 16HBE14o- line of human bronchial epithelial cells, when embedded in alginate hydrogels crosslinked with 100 mM or 300 mM calcium chloride solution concentrations containing extracellular matrix (ECM) components, do not have altered long-term viability. When methylcellulose is added to the alginate–ECM hydrogel in a 1:1 ratio with alginate, higher concentrations of calcium chloride crosslinking solution yield a significant decrease in cell viability. Furthermore, it has been observed that higher calcium chloride crosslinking solution concentrations spatially confine cells in alginate–ECM hydrogels, but not necessarily alginate–methylcellulose–ECM hydrogels, warranting further investigation.

## Figures and Tables

**Table 1:** Reported studies employing divalent cation solutions to ionically crosslink cell-laden alginate-based hydrogels.

Hydrogel	Crosslinking Solution, Duration	Result	Reference
Alginate (1.5% *w*/*v*)	102 mM CaCl_2_; N/A	75% viability of A549 alveolar adenocarcinoma.	[10]
Alginate (1% *w*/*v*)	1% (*w*/*v*) CaCl_2_; N/A	More than 80% viability of A549, U2OS, and HepG2.	[11]
Alginate (2% *w*/*v*)	100 mM, 500 mM, 1 M CaCl_2_; 5, 10, 30 min.	As calcium chloride concentration or duration of crosslinking increases, viability and proliferation of Schwann cells decreases.	[16]
Alginate–methylcellulose (3:9% *w*/*v*)	100 mM CaCl_2_; 10 min.	65% viability of human mesenchymal stem cells.	[12]
Alginate–methylcellulose (3:9% *w*/*v*)	100 mM CaCl_2_; 10 min.	77.6% viability of human mesenchymal stem cells.	[13]
Alginate–methylcellulose (3:1, 3:3, 3:9% *w*/*v*)	3 mg mL^−1^ CaCl_2_; N/A	More than 95% viability of L929 mouse fibroblasts.	[14]
Alginate–methylcellulose (3:9% *w*/*v*)	70 mM SrCl_2_; 10 min.	60–80% viability in mouse pancreatic islets.	[15]

## Data Availability

Data used in this study will be provided upon reasonable request sent to the corresponding authors.
